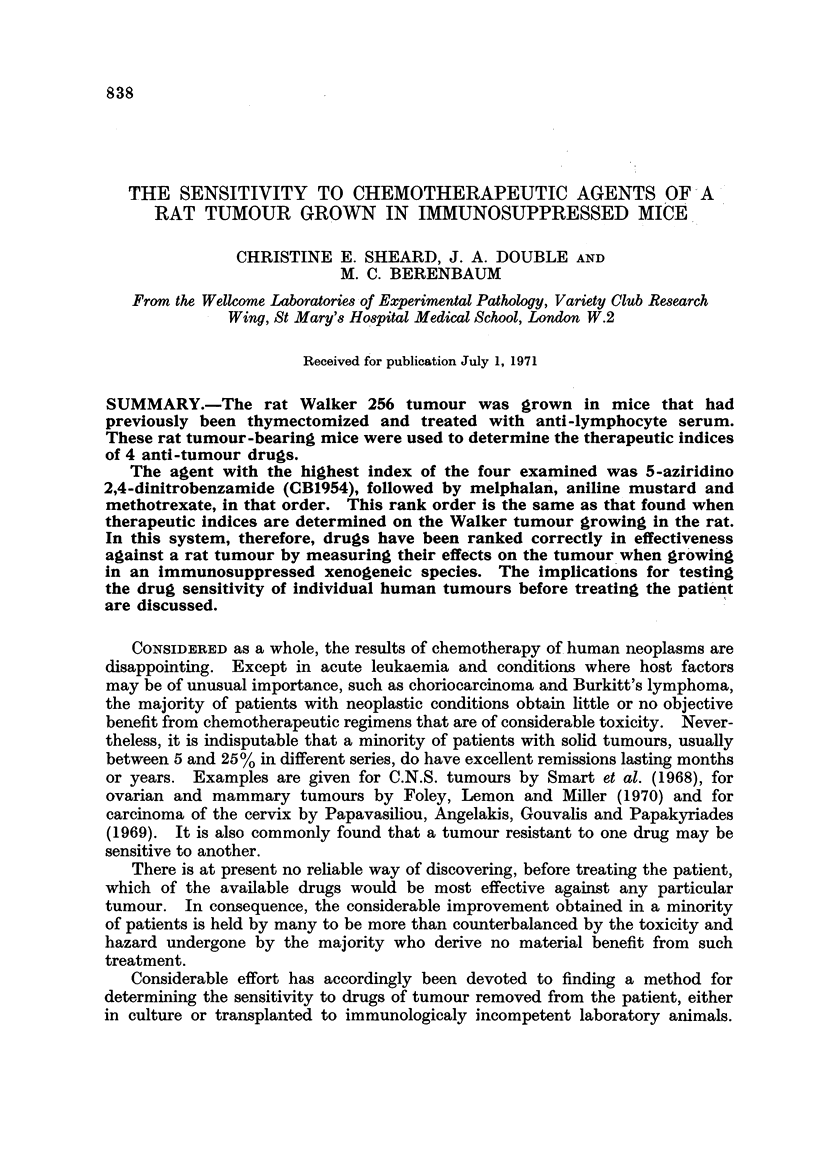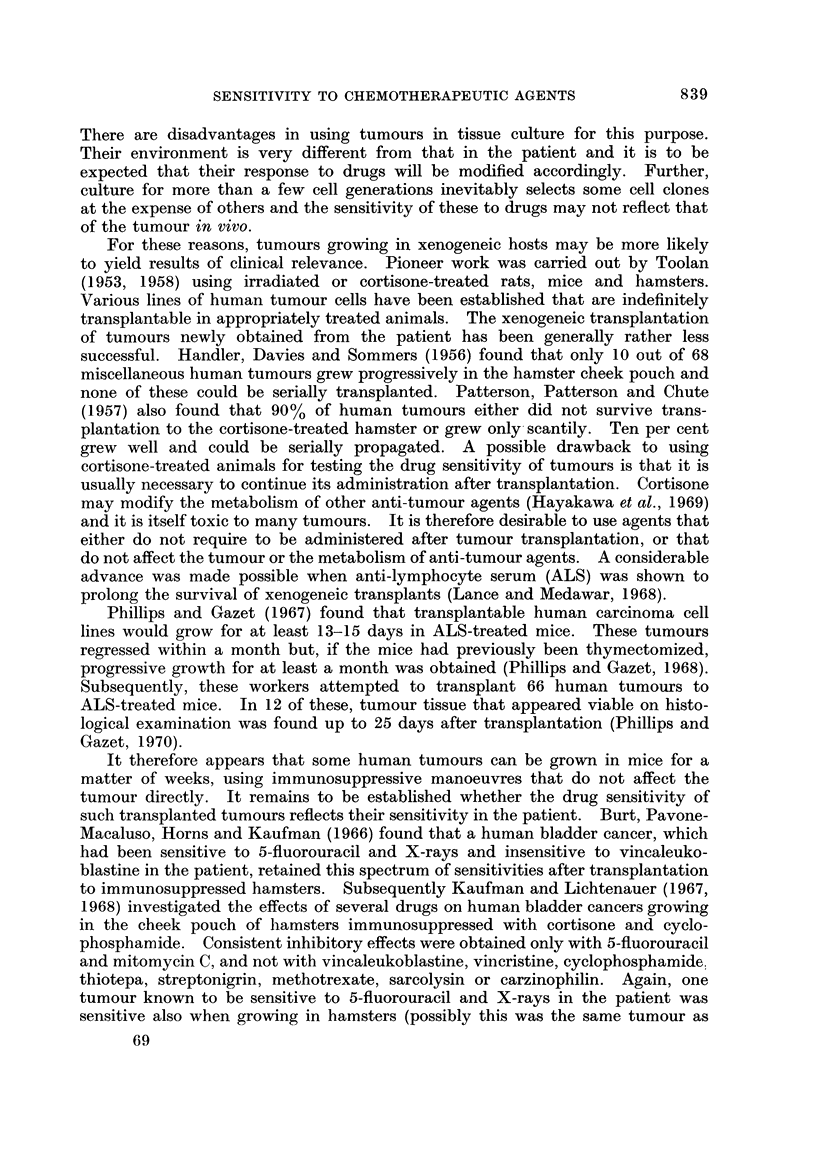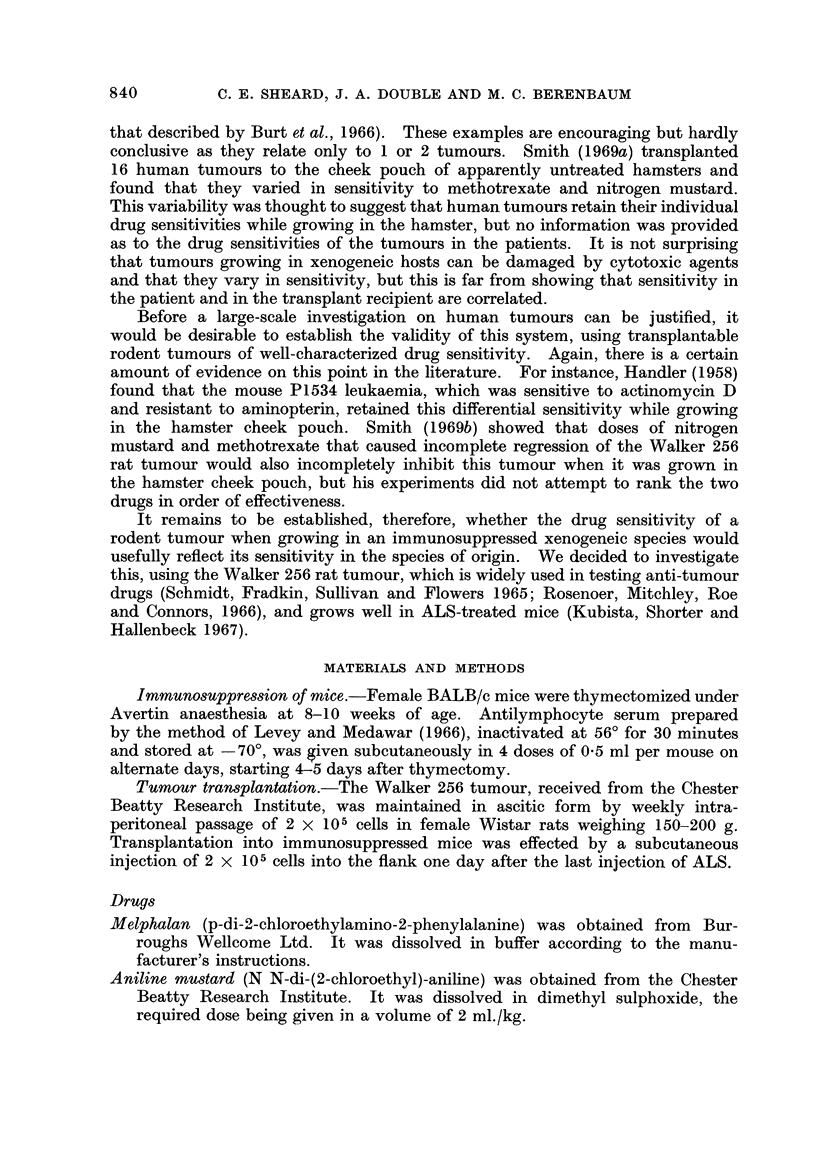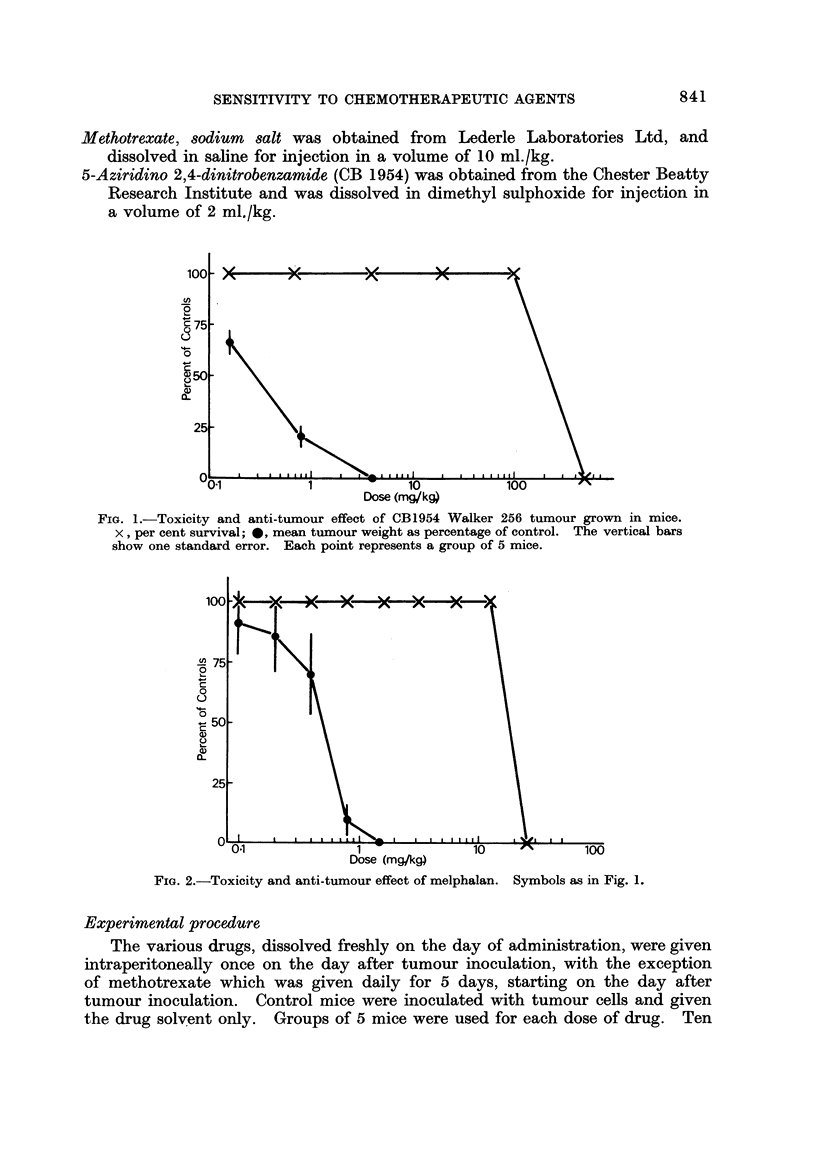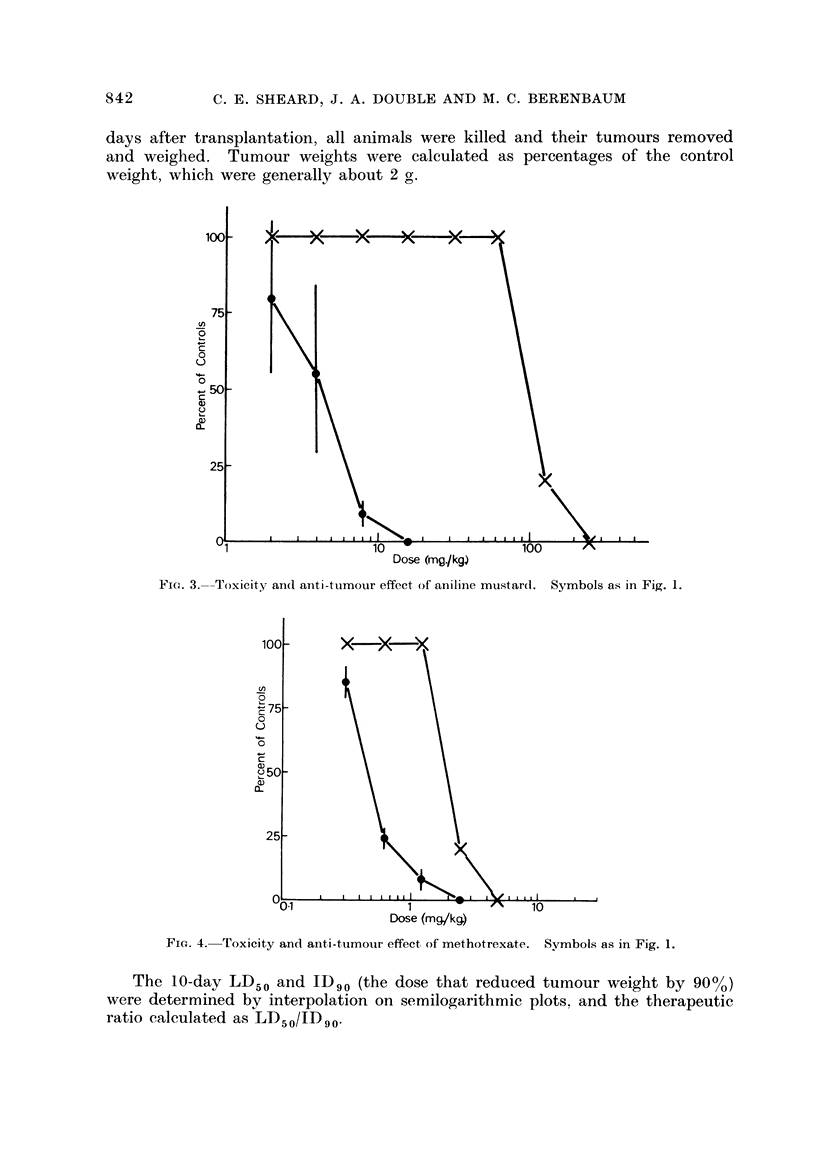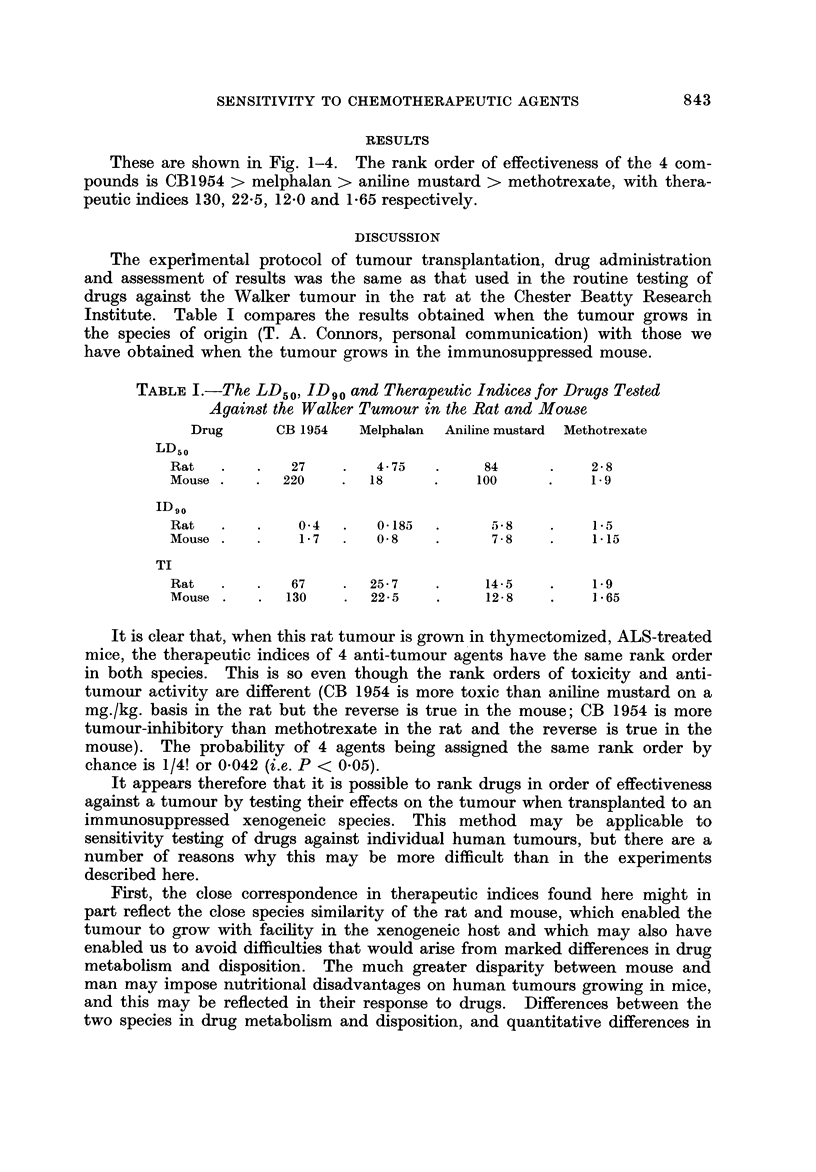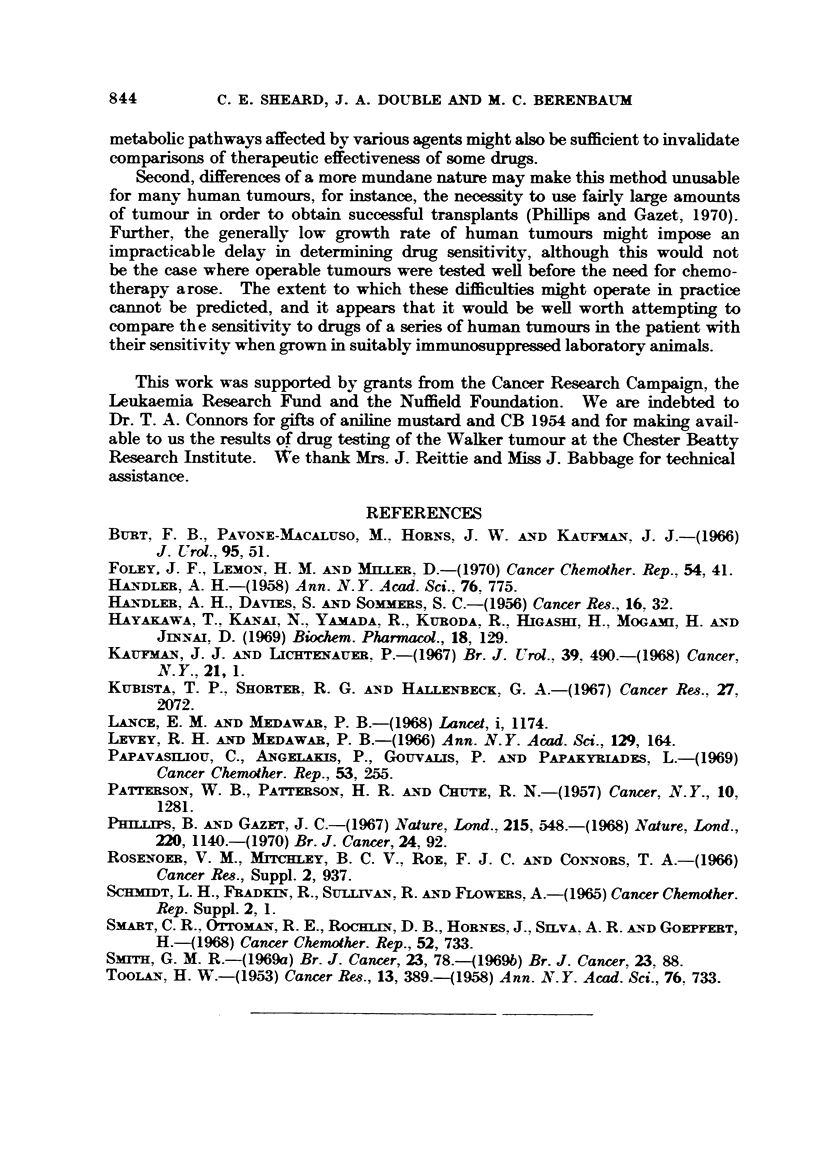# The Sensitivity to Chemotherapeutic Agents of a Rat Tumour Grown in Immunosuppressed Mice

**DOI:** 10.1038/bjc.1971.97

**Published:** 1971-12

**Authors:** Christine E. Sheard, J. A. Double, M. C. Berenbaum

## Abstract

The rat Walker 256 tumour was grown in mice that had previously been thymectomized and treated with anti-lymphocyte serum. These rat tumour-bearing mice were used to determine the therapeutic indices of 4 anti-tumour drugs.

The agent with the highest index of the four examined was 5-aziridino 2,4-dinitrobenzamide (CB1954), followed by melphalan, aniline mustard and methotrexate, in that order. This rank order is the same as that found when therapeutic indices are determined on the Walker tumour growing in the rat. In this system, therefore, drugs have been ranked correctly in effectiveness against a rat tumour by measuring their effects on the tumour when growing in an immunosuppressed xenogeneic species. The implications for testing the drug sensitivity of individual human tumours before treating the patient are discussed.


### THE SENSITIVITY TO CHEMOTHERAPEUTIC AGENTS OF A RAT TUMOUR GROWN IN IMMUNOSUPPRESSED MICE

CHRISTINE E. SHEARD, J. A. DOUBLE AND
M. C. BERENBAUM

*From the Wellcome Laboratories of Experimental Pathology, Variety Club Research Wing, St Mary's Hospital Medical School, London W.2*

Received for publication July 1, 1971

**SUMMARY.—The rat Walker 256 tumour was grown in mice that had previously been thymectomized and treated with anti-lymphocyte serum. These rat tumour-bearing mice were used to determine the therapeutic indices of 4 anti-tumour drugs.**

**The agent with the highest index of the four examined was 5-aziridino 2,4-dinitrobenzamide (CB1954), followed by melphalan, aniline mustard and methotrexate, in that order. This rank order is the same as that found when therapeutic indices are determined on the Walker tumour growing in the rat. In this system, therefore, drugs have been ranked correctly in effectiveness against a rat tumour by measuring their effects on the tumour when growing in an immunosuppressed xenogeneic species. The implications for testing the drug sensitivity of individual human tumours before treating the patient are discussed.**

CONSIDERED as a whole, the results of chemotherapy of human neoplasms are disappointing. Except in acute leukaemia and conditions where host factors may be of unusual importance, such as choriocarcinoma and Burkitt's lymphoma, the majority of patients with neoplastic conditions obtain little or no objective benefit from chemotherapeutic regimens that are of considerable toxicity. Nevertheless, it is indisputable that a minority of patients with solid tumours, usually between 5 and 25% in different series, do have excellent remissions lasting months or years. Examples are given for C.N.S. tumours by Smart *et al.* (1968), for ovarian and mammary tumours by Foley, Lemon and Miller (1970) and for carcinoma of the cervix by Papavasiliou, Angelakis, Gouvalis and Papakyriades (1969). It is also commonly found that a tumour resistant to one drug may be sensitive to another.

There is at present no reliable way of discovering, before treating the patient, which of the available drugs would be most effective against any particular tumour. In consequence, the considerable improvement obtained in a minority of patients is held by many to be more than counterbalanced by the toxicity and hazard undergone by the majority who derive no material benefit from such treatment.

Considerable effort has accordingly been devoted to finding a method for determining the sensitivity to drugs of tumour removed from the patient, either in culture or transplanted to immunologicaly incompetent laboratory animals.

There are disadvantages in using tumours in tissue culture for this purpose. Their environment is very different from that in the patient and it is to be expected that their response to drugs will be modified accordingly. Further, culture for more than a few cell generations inevitably selects some cell clones at the expense of others and the sensitivity of these to drugs may not reflect that of the tumour *in vivo*.

For these reasons, tumours growing in xenogeneic hosts may be more likely to yield results of clinical relevance. Pioneer work was carried out by Toolan (1953, 1958) using irradiated or cortisone-treated rats, mice and hamsters. Various lines of human tumour cells have been established that are indefinitely transplantable in appropriately treated animals. The xenogeneic transplantation of tumours newly obtained from the patient has been generally rather less successful. Handler, Davies and Sommers (1956) found that only 10 out of 68 miscellaneous human tumours grew progressively in the hamster cheek pouch and none of these could be serially transplanted. Patterson, Patterson and Chute (1957) also found that 90% of human tumours either did not survive transplantation to the cortisone-treated hamster or grew only scantily. Ten per cent grew well and could be serially propagated. A possible drawback to using cortisone-treated animals for testing the drug sensitivity of tumours is that it is usually necessary to continue its administration after transplantation. Cortisone may modify the metabolism of other anti-tumour agents (Hayakawa *et al.*, 1969) and it is itself toxic to many tumours. It is therefore desirable to use agents that either do not require to be administered after tumour transplantation, or that do not affect the tumour or the metabolism of anti-tumour agents. A considerable advance was made possible when anti-lymphocyte serum (ALS) was shown to prolong the survival of xenogeneic transplants (Lance and Medawar, 1968).

Phillips and Gazet (1967) found that transplantable human carcinoma cell lines would grow for at least 13–15 days in ALS-treated mice. These tumours regressed within a month but, if the mice had previously been thymectomized, progressive growth for at least a month was obtained (Phillips and Gazet, 1968). Subsequently, these workers attempted to transplant 66 human tumours to ALS-treated mice. In 12 of these, tumour tissue that appeared viable on histological examination was found up to 25 days after transplantation (Phillips and Gazet, 1970).

It therefore appears that some human tumours can be grown in mice for a matter of weeks, using immunosuppressive manoeuvres that do not affect the tumour directly. It remains to be established whether the drug sensitivity of such transplanted tumours reflects their sensitivity in the patient. Burt, Pavone-Macaluso, Horns and Kaufman (1966) found that a human bladder cancer, which had been sensitive to 5-fluorouracil and X-rays and insensitive to vincaleukoblastine in the patient, retained this spectrum of sensitivities after transplantation to immunosuppressed hamsters. Subsequently Kaufman and Lichtenauer (1967, 1968) investigated the effects of several drugs on human bladder cancers growing in the cheek pouch of hamsters immunosuppressed with cortisone and cyclophosphamide. Consistent inhibitory effects were obtained only with 5-fluorouracil and mitomycin C, and not with vincaleukoblastine, vincristine, cyclophosphamide, thiotepa, streptonigrin, methotrexate, sarcolysin or carzinophilin. Again, one tumour known to be sensitive to 5-fluorouracil and X-rays in the patient was sensitive also when growing in hamsters (possibly this was the same tumour as that described by Burt *et al.*, 1966). These examples are encouraging but hardly conclusive as they relate only to 1 or 2 tumours. Smith (1969*a*) transplanted 16 human tumours to the cheek pouch of apparently untreated hamsters and found that they varied in sensitivity to methotrexate and nitrogen mustard. This variability was thought to suggest that human tumours retain their individual drug sensitivities while growing in the hamster, but no information was provided as to the drug sensitivities of the tumours in the patients. It is not surprising that tumours growing in xenogeneic hosts can be damaged by cytotoxic agents and that they vary in sensitivity, but this is far from showing that sensitivity in the patient and in the transplant recipient are correlated.

Before a large-scale investigation on human tumours can be justified, it would be desirable to establish the validity of this system, using transplantable rodent tumours of well-characterized drug sensitivity. Again, there is a certain amount of evidence on this point in the literature. For instance, Handler (1958) found that the mouse P1534 leukaemia, which was sensitive to actinomycin D and resistant to aminopterin, retained this differential sensitivity while growing in the hamster cheek pouch. Smith (1969*b*) showed that doses of nitrogen mustard and methotrexate that caused incomplete regression of the Walker 256 rat tumour would also incompletely inhibit this tumour when it was grown in the hamster cheek pouch, but his experiments did not attempt to rank the two drugs in order of effectiveness.

It remains to be established, therefore, whether the drug sensitivity of a rodent tumour when growing in an immunosuppressed xenogeneic species would usefully reflect its sensitivity in the species of origin. We decided to investigate this, using the Walker 256 rat tumour, which is widely used in testing anti-tumour drugs (Schmidt, Fradkin, Sullivan and Flowers 1965; Rosenoer, Mitchley, Roe and Connors, 1966), and grows well in ALS-treated mice (Kubista, Shorter and Hallenbeck 1967).

### MATERIALS AND METHODS

*Immunosuppression of mice.*—Female BALB/c mice were thymectomized under Avertin anaesthesia at 8–10 weeks of age. Antilymphocyte serum prepared by the method of Levey and Medawar (1966), inactivated at 56° for 30 minutes and stored at $-70°$, was given subcutaneously in 4 doses of 0·5 ml per mouse on alternate days, starting 4–5 days after thymectomy.

*Tumour transplantation.*—The Walker 256 tumour, received from the Chester Beatty Research Institute, was maintained in ascitic form by weekly intraperitoneal passage of $2 \times 10^5$ cells in female Wistar rats weighing 150–200 g. Transplantation into immunosuppressed mice was effected by a subcutaneous injection of $2 \times 10^5$ cells into the flank one day after the last injection of ALS.

### *Drugs*

*Melphalan* (p-di-2-chloroethylamino-2-phenylalanine) was obtained from Burroughs Wellcome Ltd. It was dissolved in buffer according to the manufacturer's instructions.

*Aniline mustard* (N N-di-(2-chloroethyl)-aniline) was obtained from the Chester Beatty Research Institute. It was dissolved in dimethyl sulphoxide, the required dose being given in a volume of 2 ml./kg.

*Methotrexate, sodium salt* was obtained from Lederle Laboratories Ltd, and dissolved in saline for injection in a volume of 10 ml./kg.

5-*Aziridino* 2,4-*dinitrobenzamide* (CB 1954) was obtained from the Chester Beatty Research Institute and was dissolved in dimethyl sulphoxide for injection in a volume of 2 ml./kg.

FIG. 1.—Toxicity and anti-tumour effect of CB1954 Walker 256 tumour grown in mice. ×, per cent survival; ●, mean tumour weight as percentage of control. The vertical bars show one standard error. Each point represents a group of 5 mice.

FIG. 2.—Toxicity and anti-tumour effect of melphalan. Symbols as in Fig. 1.

*Experimental procedure*

The various drugs, dissolved freshly on the day of administration, were given intraperitoneally once on the day after tumour inoculation, with the exception of methotrexate which was given daily for 5 days, starting on the day after tumour inoculation. Control mice were inoculated with tumour cells and given the drug solvent only. Groups of 5 mice were used for each dose of drug. Ten days after transplantation, all animals were killed and their tumours removed and weighed. Tumour weights were calculated as percentages of the control weight, which were generally about 2 g.

Fig. 3.—Toxicity and anti-tumour effect of aniline mustard. Symbols as in Fig. 1.

Fig. 4.—Toxicity and anti-tumour effect of methotrexate. Symbols as in Fig. 1.

The 10-day $LD_{50}$ and $ID_{90}$ (the dose that reduced tumour weight by 90%) were determined by interpolation on semilogarithmic plots, and the therapeutic ratio calculated as $LD_{50}/ID_{90}$.

### RESULTS

These are shown in Fig. 1–4. The rank order of effectiveness of the 4 compounds is CB1954 > melphalan > aniline mustard > methotrexate, with therapeutic indices 130, 22·5, 12·0 and 1·65 respectively.

### DISCUSSION

The experimental protocol of tumour transplantation, drug administration and assessment of results was the same as that used in the routine testing of drugs against the Walker tumour in the rat at the Chester Beatty Research Institute. Table I compares the results obtained when the tumour grows in the species of origin (T. A. Connors, personal communication) with those we have obtained when the tumour grows in the immunosuppressed mouse.

TABLE I.—*The $LD_{50}$, $ID_{90}$ and Therapeutic Indices for Drugs Tested Against the Walker Tumour in the Rat and Mouse*

| Drug | CB 1954 | Melphalan | Aniline mustard | Methotrexate |
|---|---|---|---|---|
| $LD_{50}$ | | | | |
| Rat | 27 | 4·75 | 84 | 2·8 |
| Mouse | 220 | 18 | 100 | 1·9 |
| $ID_{90}$ | | | | |
| Rat | 0·4 | 0·185 | 5·8 | 1·5 |
| Mouse | 1·7 | 0·8 | 7·8 | 1·15 |
| TI | | | | |
| Rat | 67 | 25·7 | 14·5 | 1·9 |
| Mouse | 130 | 22·5 | 12·8 | 1·65 |

It is clear that, when this rat tumour is grown in thymectomized, ALS-treated mice, the therapeutic indices of 4 anti-tumour agents have the same rank order in both species. This is so even though the rank orders of toxicity and anti-tumour activity are different (CB 1954 is more toxic than aniline mustard on a mg./kg. basis in the rat but the reverse is true in the mouse; CB 1954 is more tumour-inhibitory than methotrexate in the rat and the reverse is true in the mouse). The probability of 4 agents being assigned the same rank order by chance is 1/4! or 0·042 (*i.e.* $P < 0{\cdot}05$).

It appears therefore that it is possible to rank drugs in order of effectiveness against a tumour by testing their effects on the tumour when transplanted to an immunosuppressed xenogeneic species. This method may be applicable to sensitivity testing of drugs against individual human tumours, but there are a number of reasons why this may be more difficult than in the experiments described here.

First, the close correspondence in therapeutic indices found here might in part reflect the close species similarity of the rat and mouse, which enabled the tumour to grow with facility in the xenogeneic host and which may also have enabled us to avoid difficulties that would arise from marked differences in drug metabolism and disposition. The much greater disparity between mouse and man may impose nutritional disadvantages on human tumours growing in mice, and this may be reflected in their response to drugs. Differences between the two species in drug metabolism and disposition, and quantitative differences in metabolic pathways affected by various agents might also be sufficient to invalidate comparisons of therapeutic effectiveness of some drugs.

Second, differences of a more mundane nature may make this method unusable for many human tumours, for instance, the necessity to use fairly large amounts of tumour in order to obtain successful transplants (Phillips and Gazet, 1970). Further, the generally low growth rate of human tumours might impose an impracticable delay in determining drug sensitivity, although this would not be the case where operable tumours were tested well before the need for chemotherapy arose. The extent to which these difficulties might operate in practice cannot be predicted, and it appears that it would be well worth attempting to compare the sensitivity to drugs of a series of human tumours in the patient with their sensitivity when grown in suitably immunosuppressed laboratory animals.

This work was supported by grants from the Cancer Research Campaign, the Leukaemia Research Fund and the Nuffield Foundation. We are indebted to Dr. T. A. Connors for gifts of aniline mustard and CB 1954 and for making available to us the results of drug testing of the Walker tumour at the Chester Beatty Research Institute. We thank Mrs. J. Reittie and Miss J. Babbage for technical assistance.

### REFERENCES

Burt, F. B., Pavone-Macaluso, M., Horns, J. W. and Kaufman, J. J.—(1966) *J. Urol.*, **95**, 51.

Foley, J. F., Lemon, H. M. and Miller, D.—(1970) *Cancer Chemother. Rep.*, **54**, 41.

Handler, A. H.—(1958) *Ann. N.Y. Acad. Sci.*, **76**, 775.

Handler, A. H., Davies, S. and Sommers, S. C.—(1956) *Cancer Res.*, **16**, 32.

Hayakawa, T., Kanai, N., Yamada, R., Kuroda, R., Higashi, H., Mogami, H. and Jinnai, D. (1969) *Biochem. Pharmacol.*, **18**, 129.

Kaufman, J. J. and Lichtenauer, P.—(1967) *Br. J. Urol.*, **39**, 490.—(1968) *Cancer, N.Y.*, **21**, 1.

Kubista, T. P., Shorter, R. G. and Hallenbeck, G. A.—(1967) *Cancer Res.*, **27**, 2072.

Lance, E. M. and Medawar, P. B.—(1968) *Lancet*, i, 1174.

Levey, R. H. and Medawar, P. B.—(1966) *Ann. N.Y. Acad. Sci.*, **129**, 164.

Papavasiliou, C., Angelakis, P., Gouvalis, P. and Papakyriades, L.—(1969) *Cancer Chemother. Rep.*, **53**, 255.

Patterson, W. B., Patterson, H. R. and Chute, R. N.—(1957) *Cancer, N.Y.*, **10**, 1281.

Phillips, B. and Gazet, J. C.—(1967) *Nature, Lond.*, **215**, 548.—(1968) *Nature, Lond.*, **220**, 1140.—(1970) *Br. J. Cancer*, **24**, 92.

Rosenoer, V. M., Mitchley, B. C. V., Roe, F. J. C. and Connors, T. A.—(1966) *Cancer Res.*, Suppl. **2**, 937.

Schmidt, L. H., Fradkin, R., Sullivan, R. and Flowers, A.—(1965) *Cancer Chemother. Rep.* Suppl. **2**, 1.

Smart, C. R., Ottoman, R. E., Rochlin, D. B., Hornes, J., Silva, A. R. and Goepfert, H.—(1968) *Cancer Chemother. Rep.*, **52**, 733.

Smith, G. M. R.—(1969*a*) *Br. J. Cancer*, **23**, 78.—(1969*b*) *Br. J. Cancer*, **23**, 88.

Toolan, H. W.—(1953) *Cancer Res.*, **13**, 389.—(1958) *Ann. N.Y. Acad. Sci.*, **76**, 733.